# Development of a psychological frailty index: results from the China health and retirement longitudinal study

**DOI:** 10.3389/fpsyg.2025.1495733

**Published:** 2025-02-11

**Authors:** Jinlong Zhao, Justina Yat Wa Liu, Daniel Fernández, Stefanos Tyrovolas

**Affiliations:** ^1^School of Nursing, Faculty of Health and Social Sciences, The Hong Kong Polytechnic University, Kowloon, Hong Kong SAR, China; ^2^Research Institute for Smart Ageing, PolyU Institutional Research Archive, The Hong Kong Polytechnic University, Kowloon, Hong Kong SAR, China; ^3^Department of Statistics and Operations Research (DEIO). Universitat Politècnica de Catalunya BarcelonaTech (UPC), Barcelona, Spain; ^4^Department of Nutrition and Food Studies, George Mason University, Fairfax, VA, United States; ^5^Research, Innovation and Teaching Unit, Parc Sanitari Sant Joan de Déu, Sant Boi de Llobregat, Spain; ^6^Instituto de Salud Carlos III, Centro de Investigación Biomédica en Red de Salud Mental, Centro de Investigación Biomédica en Red de Salud Mental (CIBERSAM), Madrid, Spain

**Keywords:** frailty, psychological frailty, CHARLS database, scale development, gerontology

## Abstract

**Objective:**

Psychological frailty, an emerging concept, lacks a standardized definition, measuring instrument, and empirical evidence in Asian (especially Chinese) populations. An effective instrument to measure psychological frailty should be urgently developed. Therefore, this study aimed to develop and initially validate a Psychological Frailty Index (PFI) based on the China Health and Retirement Longitudinal Study (CHARLS). The study assessed the applicability of the PFI to adverse health outcomes as a secondary aim.

**Results:**

Factor analysis of the 15-item PFI extracted four factors of psychological frailty (psychological distress, cognitive decline, physical vulnerability, and memory decline). The PFI demonstrated satisfactory internal consistency (Cronbach’s alpha = 0.764) and criterion validity (rho = 0.806). Psychological frailty was significantly associated with lower life expectancy (odds ratio [OR] 1.98, 95% confidence interval [CI] 1.71–2.29), higher outpatient treatments (1.25, 1.03–1.51), and increased hospitalization (1.45, 1.22–1.74).

**Conclusion:**

The PFI could be a reliable instrument for identifying psychological frailty. The PFI is a novel tool that measures health indicators of older adults at risk of increased psychological vulnerability, but it requires further validation.

## 1 Introduction

Frailty is an age-associated state with greater vulnerability to adverse health outcomes and a decreased ability to preserve homeostasis following stressful events for older adults (Clegg et al., 2013; Dent et al., 2019). It is also considered multidimensional because of its physical, cognitive, psychological, and social domains (Fried et al., 2021; Rockwood and Mitnitski, 2007). Therefore, it has been contended that the definitions and measurements of frailty should include multiple domains, such as physical, cognitive, psychological, and social frailty (Khezrian et al., 2017; Pilotto et al., 2020).

Psychological frailty has emerged as a novel concept. Some researchers have explored its definitions and measurements. Psychological frailty has been operationally defined as the co-presence of emotional problems and loneliness (De Witte et al., 2013). Similar to physical frailty, as Fitten (2015) argues, psychological frailty should be defined as a decrease in individuals’ emotional or cognitive resilience triggered by their exposure to stressful events. Further, psychological frailty has been considered the coexistence of physical frailty and depression (Shimada et al., 2019), indicating the necessity of defining it with other domains (i.e., physical frailty). However until today, psychological frailty lacks a unified and consistently-accepted conceptual framework (Zhao et al., 2023).

Although several measures have been proposed to measure psychological frailty (e.g., the Tilburg Frailty Indicator [TFI]), most have not articulated a clear gold standard for measuring it (Zhao et al., 2023). Further, the multiple components of psychological frailty and the validation of its architecture (Zhao et al., 2023) have not been considered. Specifically, the TFI was developed to measure multidimensional frailty; however, it is often used to measure psychological frailty. It includes three domains: physical, psychological, and social (Gobbens et al., 2010a; Gobbens et al., 2010b). Only the physical domain has shown adequate internal consistency (Gobbens and Uchmanowicz, 2021), while the psychological domain has been reported to be insufficiently comprehensive (Zamora-Sánchez et al., 2022). Nevertheless, existing studies have often used TFI to measure psychological frailty (Zhao et al., 2023). Several researchers have proposed the combined utilization of the Fried frailty phenotype (FFP) and mental health scales (e.g., the Center for Epidemiologic Studies Depression Scale; CES-D) (Rietman et al., 2018; Shimada et al., 2019). However, this approach requires further validation because there is no evidence as to whether the attributes of psychological frailty or simply the comorbid state of frailty and mental disorders is measured (Zhao et al., 2023).

To the best of our knowledge, there is no current methodological consensus on creating a psychological frailty index (PFI), and no standardized measures have been developed using the China Health and Retirement Longitudinal Study (CHARLS). Nevertheless, previous studies have reported that the cumulative deficit model (the Frailty Index [FI]) is commonly considered an appropriate method for constructing frailty-associated indices (Rockwood, 2016; Zhao et al., 2023). One of the strengths of this model is that it involves diverse variables across multiple dimensions, which facilitate the development of a PFI (Theou et al., 2023). A recent scoping review (Zhao et al., 2023) highlighted that existing variables used to define psychological frailty differ significantly and are derived from different dimensions. Another strength of the cumulative deficit model is that it can yield continuous scores for psychological frailty instead of using the three categories of frailty phenotypes (Searle et al., 2008). Therefore, a PFI based on the cumulative deficit model may more accurately predict adverse health outcomes (Cesari et al., 2014).

Although it is controversial (Zhao et al., 2023), there are two main reasons to include physical vulnerability (physical frailty) when using a cumulative deficit model to generate a psychological frailty index. First, as part of (multidimensional) frailty (Gobbens et al., 2024), psychological frailty is bound to have the attributes of frailty. If not, it is challenging to differentiate “psychological frailty” from other similar psychological terms, such as psychological distress (Zhao et al., 2023). The core attribute of frailty can be vulnerability at the physiological and physical level (Dent et al., 2019; Fried et al., 2021; Khezrian et al., 2017). Therefore, coupled with physical vulnerability, it is a distinctive characteristic of psychological frailty that differentiates it from similar psychological terms. Second, physical frailty has been integrated into the conceptual framework of psychological frailty in a recently published work by Shimada et al. (2019). Other similar studies have demonstrated the integration of physical vulnerability (physical frailty) and cognitive problems to construct cognitive frailty (Kelaiditi et al., 2013; Sugimoto et al., 2022), reinforcing the view that physical vulnerability is integrated into the cognitive frailty framework. For the above reasons, we have considered adding physical vulnerability in our PFI construction.

A new instrument for psychological frailty is likely to facilitate the understanding of its essential properties, including potential opportunities to attenuate its course; however, such an instrument is lacking.

## 2 Methods

### 2.1 Aim, design, and setting

This study primarily aimed to create a PFI using the CHARLS, considering the FI approach. Its reliability and validity, including classification accuracy and applicability, were evaluated (partially) as secondary aims.

### 2.2 Data source and study population

The CHARLS is an ongoing, nationally-representative survey of the Chinese population covering health information, economic data, and health records (Zhao et al., 2020). A multi-stage probability-proportional-to-size sampling procedure was used to identify participants at baseline (2011–2012). Follow-up visits were conducted every 2 or 3 years and included Wave 2 (2012–2013), Wave 3 (2015), Wave 4 (2018), and Wave 5 (2021) visits. Ethical approval was granted by the Institutional Review Board of Peking University.

This study adopted a cross-sectional design and used CHARLS Wave 4 (2018) data. This study selected the sample in Wave 4 based on three criteria: (1) participants aged ≥65 years (Clegg et al., 2013); (2) participants who responded to psychological-frailty-related variables with less than 5% missing values (Theou et al., 2023); and (3) those who did not have missing values or who did not respond with “refuse to answer,” “not assessed,” or “do not know” (Mutz et al., 2021). The sample selection is shown in Supplementary Figure S1.

To better evaluate the PFI’s performance, a random sample (about 30%) was taken and partitioned. Creating a PFI using a relatively separate random sample addressed potential bias in relation to specific characteristics by randomly extracting data (Krstajic et al., 2014). Furthermore, the PFI could be evaluated using unseen and broader data to estimate its actual performance because the total sample was more extensive. After partitioning, the initial validation analyses used the total sample, facilitating a more efficient use of data (de Rooij and Weeda, 2020). Using a total sample rather than an “uncontaminated” sample was necessary because this study did not have a machine-learning design and aimed to evaluate the applicability of the PFI under specific health outcomes.

### 2.3 PFI

This study created the PFI by adapting standardized procedures outlined by Searle et al. (2008) and Tocchi et al. (2014).

#### 2.3.1 Selecting variables for the PFI

Searle et al. (2008) recommended five criteria for selecting variables for a physical FI: (1) the variables must be deficits associated with the health status; (2) the prevalence of a deficit must generally increase with age; (3) chosen deficits must not saturate too early; (4) when candidate deficits are considered as a group, the deficits that comprise an FI must cover a range of systems; and (5) if a single FI is to be used serially on the same individuals, the variables that comprise the FI need to be the same from one iteration to the next. This study partially adhered to these criteria because the PFI is not precisely equivalent to a physical FI. Specifically, the first criterion was strictly adhered to; all potential variables in this study were associated with the health status. The second criterion was only partially fulfilled because the nature of psychological frailty makes it difficult for its subjective variables to accurately indicate changes in these variables with age. As for the third criterion, not all variables were oversaturated in the early stages of aging. The fourth and fifth criteria were adopted to facilitate the selection of variables. Accordingly, 35 potential variables were screened as initial PFI items (Supplementary Table S1).

#### 2.3.2 Generating initial items for the PFI

These variables were then re-coded. For non-binary items, recoding was conducted to grade the ranking into a value between 0 (not present) and 1 (present), similar to the scoring strategy for an FI (Huang et al., 2022; Searle et al., 2008). Four- or five-level Likert-scale items derived from self-reported questions were re-coded as scores from 0 to 1 based on differing response levels in an equally spaced manner (e.g., very = 1, quite often = 0.75, somewhat = 0.5, a little = 0.25, and none = 0) (Supplementary Table S1). These re-coded levels were determined following previous studies (Mutz et al., 2022; Tyrovolas et al., 2018) and Searle et al. (2008) standard methodology on the creation of a frailty index.

#### 2.3.3 Evaluating the initial items

Categorical principal component analysis (CatPCA) (Linting et al., 2007; Linting and van der Kooij, 2012) was used to evaluate the initial items of the PFI with a random sample (*n* = 1,290). CatPCA is a statistical technique used to reduce dimensionality when categorical variables are examined, as it is the nonlinear equivalent of the standard PCA where categorical variables are quantified while the number of dimensions in the data is reduced (Casacci, 2023; Linting et al., 2007).

#### 2.3.4 Generating the scoring system and the cut-off value

A composed PFI was generated as the proportion of items present in an older adult out of the total number of items, similar to the construction of a physical FI (Searle et al., 2008). The entire score was derived using a non-weighted calculation ranging from 0 to 1, with a higher score indicating a more severe degree of psychological frailty. As no prior studies have investigated the optimal cut-off value or established a gold standard for psychological frailty (Zhao et al., 2023), the optimal cut-off value was determined using the Youden index. Additionally, population tertiles were also used to establish cut-off values, as many similar studies related to the FI have used population tertiles or quartiles (Clegg et al., 2016; Dunlay et al., 2014).

### 2.4 Additional calibration instruments

The TFI, developed by Gobbens et al. (2010b), is used to assess multidimensional frailty. This instrument has 15 items covering physical, psychological, and social domains. The psychological domain is measured using four items related to coping, depression, anxiety, and cognition. A score > 5 indicates frailty (Gobbens et al., 2010b). The TFI has been validated among older Chinese adults (Dong et al., 2017; Si et al., 2021). This study followed the TFI based on 14 relevant variables derived from Wave 4. The variable related to weight was not applied owing to data unavailability (Supplementary Table S2).

The 10-item CES-D is an instrument used for assessing depression worldwide. The full version consists of 20 items used to inquire how often an individual experienced depression-related symptoms in the past week (Radloff, 1977). The 10-item version, developed by Andresen et al. (1994), has been validated in Chinese populations with a recommended cut-off value of 10 (Fu et al., 2022; Yang et al., 2018) and used as a routine item in the CHARLS questionnaire.

Owing to the absence of a gold standard, the TFI and the 10-item CES-D were chosen as the “quasi-gold” standards for validating the PFI. The TFI was selected because it is a widely used measure of multidimensional frailty (Zamora-Sánchez et al., 2022), and existing studies frequently use its subscale (psychological domain) to assess psychological frailty (Zhao et al., 2023). The 10-item CES-D was adopted as it is a standard measure of depression (Schein and Koenig, 1997), which is considered a core construct of psychological frailty (Zhao et al., 2023).

### 2.5 Adverse health outcomes

This study considered three outcomes. Lower life expectancy was defined as having an individual’s lower subjective life expectancy; that is, participants were less likely to assume that they would live 10–15 years at the time of measurement. Missing values, including “do not know,” were contained in this outcome. While item-level missingness (Newman, 2014) was involved, their effect on the results was likely to be small. This outcome was retained because it was a critical variable for representing the structure of subjective life expectancy. Similar to missing-data treatments utilized in previous studies (Denman et al., 2018; Mirzaei et al., 2022), missing values were treated as neutral responses because they had no real value. This outcome was transformed, and the original five responses were dichotomized into “No” (0) and “Yes” (1). This reduction in responses considerably simplified the outcome’s categorical hierarchy, explicitly improving the interpretability of this outcome. That is, this outcome is more understandable and interpreted in a way that aligns more with the readers’ categorical expectations of this outcome (Wittink and Bayer, 1994). In addition, we ran an ordinal regression analysis using this outcome (lower life expectancy rated using a five-point Likert), and the results remain consistent with the binary analysis for the total sample as well as for subgroups (data not shown in the text). Outpatient treatment was measured using the question, “In the last month, have you visited a public hospital, private hospital, public health center, clinic, health worker’s or doctor’s practice, or been visited by a health worker or doctor for outpatient care (not including physical examination)?” (Yes/No). Hospitalization was assessed using the question, “Have you received inpatient care in the past year?” (Yes/No). The rationale for selecting these three variables as outcomes included: (1) increased potential risks of these outcomes emerging in older adults with psychological frailty (Calciolari and Luini, 2023; Yong et al., 2021; Zhang et al., 2018); and (2) the previous use of these variables in relevant studies (Daniels et al., 2012; Gobbens et al., 2012).

### 2.6 Covariates

Covariates included age, sex, the total number of chronic diseases, financial status (additional income: “Did you receive any wage and bonus income (excluding pensions) in the past year?”; Yes/No), highest educational qualification (four levels: being illiterate or not completing primary school, receiving home or primary school education; secondary school education; and higher education), physical disability (Yes/No), light physical activity: walking (e.g., at home, and for exercise or recreation) at least 10 min every week (Yes/No), moderate physical activity: allowing you to breathe faster than usual (e.g., doing Tai Chi) at least 10 min every week (Yes/No), and smoking status: “Have you ever chewed tobacco, smoked a pipe, smoked self-rolled cigarettes, or smoked cigarettes/cigars?” (Yes/No). Previous research has demonstrated that these covariates have a substantial relationship with psychological and general frailty (Tyrovolas et al., 2018; Uchmanowicz et al., 2022; Zhao et al., 2023). For example, age is a critical covariate in psychological frailty as it is directly associated with physiological declines of the brain, increased vulnerability, and multiple adverse health outcomes.

### 2.7 Statistical analysis

Descriptive statistics were used to characterize the basic features of the sample. Frequencies and percentages (%) were reported for categorical variables, and the mean ± standard deviation (SD) (or median ± interquartile range [IQR]) was reported for continuous variables. Spearman’s *ρ* coefficient was used to assess the criterion-related validity, while the Kolmogorov–Smirnov test was used to assess the distribution normality of the PFI. CatPCA with varimax and Kaiser normalization was performed to explore the multidimensional nature of psychological frailty and select appropriate variables. This study relied on both Kaiser’s criterion and Comrey’s (1973) criterion: (1) all factors with an eigenvalue above one were retained; (2) variables with loadings higher than 0.25 were used to explain the components obtained; and (3) the variance accounted for (VAF) of each component was assessed using the following criteria: 0.10, poor; 0.20, fair; 0.30, good; 0.40, very good; and 0.50, excellent (Linting and van der Kooij, 2012; Saukani and Ismail, 2019). Cronbach’s alpha value ≥0.70 is good, while a value ≥0.80 is excellent (Flora, 2020; Kalkbrenner, 2023; Kline, 2000; Taylor, 2021). Confirmatory factor analysis (CFA) was conducted to validate the hypothetical construction of the PFI using maximum likelihood estimation. The criteria for the goodness-of-fit indices are presented in Table 2. Receiver operating characteristic (ROC) curve analysis was used to identify cutoff values and assess the classification accuracy. Binary logistic regression models were used to examine associations between the odds ratios (ORs) of the PFI and lower (subjective) life expectancy, outpatient treatment, and hospitalization after adjusting for all covariates. Analyses were further stratified by sex and age (young-old: 65–74 years; old-old: ≥75 years) according to the age criterion of Au et al. (2017). Binary regression models and ordinal regression models were implemented using the *glm* function from the package *stats* and the *polr* function from the package *mass* of R statistical software, respectively. Statistical significance was set at a two-tailed *p*-value <0.05. Statistical analyses were performed on R version 4.3.2 (R Foundation for Statistical Computing, Vienna, Austria), SPSS (version 27.0; SPSS Inc., Chicago, IL, USA) and AMOS (version 25.0; SPSS Inc., Chicago, IL, USA) software.

## 3 Results

### 3.1 Basic characteristics of participants

In Wave 4, 12,137 (61.25%) of 19,817 participants did not meet the age criteria. A further 108 participants (0.55%) were excluded owing to missing outcome variables, leaving 3,934 participants in the total sample. Of these, 1,290 (33.3%) were treated as random samples (Supplementary Figure S1). Of the total sample, more than half (53.6%, *n* = 2,108) were males. The mean age was 71.04 (SD = 5.24). In total, 1,630 (41.4%) were psychologically frail (PFI score > 0.3083 used as psychological frailty cut-off point based on youden index methodology see section 3.3 for more detail). Participants with psychological frailty were older in both females (mean 71.10 years [SD: 5.28] vs. 70.32 years [4.92], respectively) and males (71.95 [5.58] vs. 71.01 [5.18], respectively). As for participants with psychological frailty, multimorbid diseases are reported more among females (mean 3.23 [SD: 2.12] vs. 2.39 [1.81], respectively) than among males (3.23 [2.11] vs. 2.17 [1.75], respectively) (Supplementary Table S3).

### 3.2 CatPCA results

CatPCA results suggested psychological frailty had a four-factor structure, including 15 variables out of the 35 initial ones, and explaining 57.39% of the total variance (Table 1). Specifically, the eigenvalue of the first factor was 2.883, indicating that it explained 19.219% (2.883/15) of variance. The eigenvalues of the remaining three factors were 2.09, 2.021, and 1.616, representing a VAF share of approximately 38%. Based on the included 15 items grouping, these four factors were named as psychological distress, cognitive decline, physical vulnerability, and memory decline.

**Table 1:** Factor loadings and eigenvalues of the psychological frailty index (PFI).

	Names	Factor				VAF
		1	2	3	4	
PFI1	Depression	0.843				0.731
PFI18	Anxiety	0.763				0.598
PFI13	Attentional problem	0.751				0.577
PFI25	Powerlessness	0.743				0.616
PFI20	Loneliness	0.596				0.416
PFI7	Disorientation: checking the state		0.769			0.596
PFI4	Disorientation: checking year		0.701			0.518
PFI8	Disorientation: checking county		0.697			0.506
PFI5	Disorientation: checking season		0.679			0.468
PFI30	Decreased strength			0.712		0.540
PFI28	Physical exhaustion			0.704		0.533
PFI27	Limited physical activity			0.657		0.463
PFI29	Functional limitations			0.645		0.436
PFI17	Memory impairment				0.890	0.805
PFI31	Memory loss				0.889	0.806
						Total
Cronbach’s alpha		0.748	0.601	0.659	0.456	0.947
Eigenvalue		2.883	2.09	2.021	1.616	8.609
Variance explained (%)		19.219	13.931	13.473	10.77	57.394

VAF, variance accounted for Total Cronbach’s alpha is based on the total eigenvalue. Rotation method: varimax with Kaiser normalization.

### 3.3 Cut-off values of the PFI

The maximum value of the Youden index (*J* = sensitivity+specificity-1) was 0.667 for identifying psychological frailty, indicating that the optimal cut-off value was 0.3083 (sensitivity = 83.5%; specificity = 83.2%). As aforementioned in methodology, PFI ranges from 0 to 1. Both sensitivity and specificity were > 70%, indicating that 0.3083 was adequate for identifying psychological frailty (Supplementary Figure S3A). Additionally, we also used population’s tertiles to assess the cut-off values for the three types of psychological frailty— the lowest tertile (≤0.20) indicated a robust state; the middle (>0.20 to <0.35) indicated pre-psychological frailty; and the highest (≥ 0.35) indicated psychological frailty.

### 3.4 Reliability and validity assessment results

#### 3.4.1 Reliability

Cronbach’s alpha for the PFI was 0.764 (95% confidence interval [CI]: 0.753–0.775). Since it is usually challenging to fulfill Cronbach’s *α* coefficient assumptions (Flora, 2020; McNeish, 2018), other alternative indicators were also reported. The PFI performed well, with a total omega coefficient of 0.757 (0.746–0.775), greatest lower bound (GLB) of 0.817, and coefficient H of 0.834.

#### 3.4.2 Criterion-related validity

Spearman’s *ρ* coefficient between the PFI and TFI was 0.806 (*p* < 0.001). A high correlation was also observed between the PFI and the 10-item CES-D (rho = 0.773, *p* < 0.001). Spearman’s ρ coefficient was greater than 0.70, indicating good criterion-related validity (Spearman, 1904). The TFI and the 10-item CES-D had good internal consistency reliability (TFI: Cronbach’s α = 0.730, 95% CI: 0.717–0.742; 10-item CES-D: 0.797, 0.788–0.806).

#### 3.4.3 Construct validity and classification accuracy

In the CFA model, when applied to the total sample, all paths of items were significantly loaded to the hypothesized factors (loading range: 0.45 to 0.78) (Supplementary Figure S2). Since characteristics of PFI may differ across genders we also applied a CFA for the subgroup of men and women, and the results showed that the PFI exhibited weak invariance and satisfied strong and strict invariance, supporting the stability of the consistent factor structure across genders (data not shown in text). In terms of the total sample model’s fitness, the following values were obtained: chi-square (χ^2^/*df*), 2.715 (< 3); standardized root mean square residual, 0.019 (*p* < 0.06), indicating a better fit to the hypothesized model; root mean square error of approximation value, 0.021 (90% CI: 0.018–0.024; < 0.06), indicating an excellent fit; and satisfactory goodness-of-fit index, 0.993 (>0.950); adjusted goodness-of-fit index, 0.989 (>0.950); normed fit index, 0.983 (>0.950); Tucker-Lewis index, 0.986 (>0.950); incremental fit index, 0.986 (>0.950); comparative fit index, 0.989 (>0.950); relative fit index, 0.978 (>0.950), and parsimony-adjusted comparative fit index, 0.740 (>0.50) (Table 2).

**Table 2:** Summary of results of goodness-of-fit indices.

Indicators	Results	Best threshold value
χ^2^/*df*	2.715	<2 or <3
RMR	0.002	The smaller the better, 0 is perfect
SRMR	0.019	< 0.06 (Good) or <0.08 (Acceptable)
RMSEA	0.021 (90% CI: 0.018 to 0.024)	< 0.06 (Good) or <0.08 (Acceptable)
GFI	0.993	>0.95
AGFI	0.989	>0.95
CN (0.05)	1,848	>200
CN (0.01)	2,038	>200
NFI	0.983	>0.95
RFI	0.978	>0.95
IFI	0.989	>0.95
TLI (NNFI)	0.986	>0.95
CFI	0.989	>0.95
PGFI	0.654	>0.50, closer to 1 the better
PCFI	0.744	>0.50
PNFI	0.74	>0.50

Criterion sources include Byrne (2006), Carmines and McIver (1981), Hoelter (1983), Hu and Bentler (1999), Kline (2016), Mulaik et al. (1989), Schreiber et al. (2006), and Wheaton et al. (1977). RMR, root mean square residual; SRMR, standardized RMR; RMSEA, root mean square error of approximation; GFI, goodness-of-fit index; AGFI, adjusted GFI; CN, Critical N; RFI, relative fit index; NFI, normed fit index; IFI, incremental fit index; TLI (NNFI), Tucker-Lewis index (non-normed fit index); CFI, comparative fit index; PGFI, Parsimony-adjusted GFI; PCFI, Parsimony-adjusted CFI; PNFI, Parsimony-adjusted NFI.

PFI classification accuracy (discriminative ability) was evaluated using the area under the ROC curve (AUC). Swets (1988) recommended that AUC values be interpreted as follows: 0.5 to 0.7 for acceptable accuracy, 0.7 to 0.9 for good accuracy, and higher than 0.9 for excellent accuracy. The AUC for multidimensional frailty was 0.913 (95% CI: 0.904–0.922), which indicated that the PFI had excellent discriminative ability and could correctly identify robust and frail participants. The AUC for categorizing diagnosed emotional or psychiatric problems was 0.677 (0.623–0.732). The AUC for classifying diagnosed memory-related diseases was 0.657 (0.620–0.694). An AUC of 0.846 (95% CI: 0.828–0.864) represented a good discriminative ability for depression (Supplementary Figure S3).

### 3.5 Evidence of applicability

The PFI’s applicability was confirmed by its significant associations with lower life expectancy (OR = 1.98, 95% CI 1.71–2.29, *p* < 0.001), outpatient treatment (1.25, 1.03–1.51, *p* = 0.022), and hospitalization (1.45, 1.22–1.74, *p* < 0.001). Young-old adults with psychological frailty increased the odds of lower life expectancy (2.01, 1.70–2.38, *p* < 0.001) and hospitalization (1.39, 1.13–1.72, *p* = 0.002). Old-old adults with psychological frailty had higher odds of lower life expectancy (1.81, 1.33–2.46, *p* < 0.001), outpatient treatment (1.53, 1.03–2.27, *p* = 0.037), and hospitalization (1.58, 1.13–2.21, *p* = 0.007). Significant associations with lower life expectancies were found in both sexes (*p* < 0.001). The adjusted odds of rating life expectancy as lower were greater for males with psychological frailty than for females with the same condition (2.09 vs. 1.92, respectively, *p* < 0.001). Males with psychologically frailty had higher odds of being hospitalized (1.60, 1.26–2.04, *p* < 0.001) (Table 3).

**Table 3:** Associations of psychological frailty with lower life expectancy, outpatient treatment, and hospitalization.

	Lower life expectancy		Outpatient treatment		Hospitalization	
Total sample (*n* = 3,934)	OR (95% CI)	*p*-value	OR (95% CI)	*p*-value	OR (95% CI)	*p*-value
Psychological frailty	1.98 (1.71–2.29)	<0.001	1.25 (1.03–1.51)	0.022	1.45 (1.22–1.74)	<0.001
Young-old (*n* = 3,006)						
Psychological frailty	2.01 (1.70–2.38)	<0.001	1.17 (0.94–1.45)	0.152	1.39 (1.13–1.72)	0.002
Old-old (*n* = 928)						
Psychological frailty	1.81 (1.33–2.46)	<0.001	1.53 (1.03–2.27)	0.037	1.58 (1.13–2.21)	0.007
Female (*n* = 1,826)						
Psychological frailty	1.92 (1.56–2.36)	<0.001	1.23 (0.94–1.61)	0.13	1.28 (0.99–1.66)	0.058
Male (*n* = 2,108)						
Psychological frailty	2.09 (1.69–2.58)	<0.001	1.26 (0.96–1.65)	0.09	1.60 (1.26–2.04)	<0.001

Outcomes: lower life expectancy is defined as the participants’ low subjective life expectancy (participants are less likely to assume they will live for 10 to 15 years from the time of measurement); outpatient treatment refers to participants’ making outpatient visits to a health care facility or receiving in-home health care services in the past month (excluding physical examinations); hospitalization indicates the hospitalization of participants in the past year. Psychological frailty was defined as PFI score >0.3083. The reference is non-psychological frailty. OR, Odds ratio. CI, confidence interval. The model was adjusted for age, sex, the total number of chronic diseases, financial status (additional income), highest education qualification, physical disability, light physical activity, moderate physical activity, and smoking status.

## 4 Discussion

### 4.1 Main findings

This study presents processes in the development of a PFI and its psychometric properties. This PFI was partially validated as an effective instrument for measuring psychological frailty. It demonstrated good performance in terms of both internal consistency, reliability, and criterion-related validity. It also showed sound construct validity and conformed to a four-factor model that included psychological distress, cognitive decline, physical vulnerability, and memory decline. Furthermore, the good discriminative ability of the PFI for identifying frailty and depression was highlighted. Its applicability was tested by showing association with lower life expectancy, higher outpatient treatment, and increased hospitalization. When stratified according to age and sex, these associations remained consistent.

To date, no cut-off value or optimal methodologies have been established for PFI (Zhao et al., 2023). This study provides the first evidence for a cutoff value in a PFI, facilitating its application in screening for psychological frailty. The PFI demonstrated satisfactory internal consistency, based on Cronbach’s alpha and other alternative indicators. The alpha value in this study was better than Cronbach’s alpha values (0.37 through 0.63) obtained for the commonly-used measure of psychological frailty (i.e., the TFI’s psychological domain) across eight studies (Gobbens and Uchmanowicz, 2021). This study also demonstrated that the PFI’s criterion-related reliability was satisfactory. Although the criteria used in this reliability analysis were not optimal, they were the most relevant options available because optimal criteria are still lacking.

The PFI also demonstrated satisfactory construct validity. CatPCA results showed that the total VAF across the four factors was over 50%, indicating that the PFI had an adequate degree of variance to explain psychological frailty. CFA results also supported the PFI’s four-factor structure, indicating its robustness and reproducibility.

This study also verified the classification accuracy (discriminative ability) of the PFI. Regarding frailty, the PFI showed excellent discriminative ability. Similarly, the PFI showed good or acceptable discriminative ability for depression, emotional and psychiatric problems, and memory-related diseases. This evidence suggests the potential of the PFI for clinical application. Nevertheless, Fan et al. (2006) emphasized the potential lack of clinical value where the relevant AUC values are <0.75. The AUC values for both doctor-diagnosed diseases were < 0.75; therefore, the value of the PFI in clinical applications requires further validation. However, the classification accuracy test in this study was intended to utilize these two doctor-diagnosed diseases as objective metrics to assess the PFI.

The findings validated the applicability of the PFI in terms of its association with a lower (subjective) life expectancy, higher outpatient treatment, and increased hospitalization. The association with lower life expectancy has not been previously explored. However, previous studies have found that frailty is associated with decreased life expectancy (Gao et al., 2023; Shamliyan et al., 2013). Moreover, several studies have identified an association between psychological frailty and mortality (Gobbens et al., 2021; Lee et al., 2022), which indirectly support this study’s findings.

This study considered life expectancy as representing an individual’s perception of longevity (O’Connell, 2011). Lower life expectancy is often associated with (psychological) distress (Bodner and Bergman, 2016; Deeg et al., 2021; Griffin et al., 2013), poorer health status (Chen et al., 2021), and limited social connectedness (Bae et al., 2017). Psychological frailty can significantly affect older adults’ perceptions of their life expectancy. Older adults with psychological frailty can hold a pessimistic view of their future health, which causes their belief that they will not survive beyond 10 to 15 years.

Our findings support the association between psychological frailty and outpatient treatment, consistent with previous studies on frailty (Fan et al., 2021; Ge et al., 2020; Li et al., 2021). Nevertheless, all these studies focused on physical or other frailty, which may differ from psychological frailty. Despite insufficient evidence, a significant positive association between psychological frailty and the number of doctor visits has been demonstrated (Calciolari and Luini, 2023). Older adults with psychological frailty tend to suffer from multiple physical and mental conditions (Gobbens et al., 2024; Mutz et al., 2022) and thus may be more likely to exhibit a higher likelihood of receiving outpatient treatment.

Additionally, this study showed that psychological frailty was associated with hospitalization, which is consistent with previous findings (Calciolari and Luini, 2023; Lee et al., 2022). Older adults with psychological frailty are more vulnerable to poor health and multimorbidity (Gobbens et al., 2024; Teo et al., 2019), such as acute exacerbations of physical or mental conditions, resulting in a greater likelihood of hospitalization. The overall pattern of findings from the age-stratified analysis was comparable to that of the principal analysis. In the sex-stratified analysis, a significant association between psychological frailty and hospitalization was found only in males. Several studies have found significant associations between frailty and hospitalization, such as the frequency of inpatient visits, hospital admissions, and the length of hospital stay (Daniels et al., 2012; Fan et al., 2021; Ge et al., 2020; Quach et al., 2023). Furthermore, compelling evidence of these associations exists in systematic reviews (Uchmanowicz et al., 2020; Vermeiren et al., 2016).

The conceptual framework of psychological frailty in our study is worthy of further discussion. Our addition of physical vulnerability in the psychological framework further highlights the complex interactions between mental and physical phenomena. Psychological frailty and physical vulnerability are inherently interrelated due to similar or shared physiological mechanisms. Specifically, psychological stress or depression can lead to inflammation or hormonal imbalances, which can directly affect physical resilience (Fried et al., 2021; Unsar and Sut, 2010; Wolkowitz et al., 2001). Similarly, physical vulnerabilities can increase the risk of psychological problems such as anxiety and depression, which can contribute to the onset of psychological frailty (Ma et al., 2023; Makizako et al., 2015; Zhao et al., 2023). This mutual relationship emphasizes that physical vulnerability can be a key factor influencing psychological frailty. Furthermore, the conceptual framework of this study highlights that critical physiological mechanisms, such as the stress response, may play a mediating role between physical vulnerability and psychological frailty. Physical vulnerability, characterized by reduced physiological reserves and increased susceptibility to stressors (Dent et al., 2019), typically interacts with psychological frailty (Gobbens et al., 2010a), including cognitive decline, mood dysregulation, and decreased memory function (Fitten, 2015; Zhao et al., 2023). Items related to physical vulnerability in our index can represent physical declines caused by aging and stress responses while also causing psychological stress. Conversely, items related to psychological components (psychological distress, cognitive and memory declines) can heighten psychological stress and simultaneously hasten physical declines. The bidirectional relationships can result in a mind–body loop of frailty, with dysregulated stress responses aggravating the mind–body vulnerability (Bot and Kuiper, 2017; Schneiderman et al., 2005). Therefore, incorporating physical vulnerability as a component into the conceptual framework of psychological frailty emphasizes the importance of addressing shared physiological mechanisms. As has been marked by previous researchers, attention should be given not only to the stress response (Pearlin, 1999) but also to other aging-associated mechanisms (Guo et al., 2022; Jin et al., 2024), such as the inflammatory response. Finally, including physical vulnerability in the PFI aligns with a previously proposed psychological frailty framework (Shimada et al., 2019). Notably, our proposed index presents a multifaceted construct of psychological frailty, thereby emphasizing its new contribution to understanding the mind–body link to frailty. Although, until today, there is no consensus on the theories and conceptual frameworks of PFI (Lameirinhas et al., 2024; Zhao et al., 2023), there are several studies that support the notion that mind–body interactions could be considered as one of the fundamental components of psychological frailty (Shimada et al., 2019; Zhao et al., 2023).

### 4.2 Strengths and limitations

This study has several strengths. This study is the first to create and initially validate a PFI. Although some studies have used several instruments to assess psychological frailty, these instruments lack sound constructs and validation. The present study addresses this gap and provides a more reliable measure of psychological frailty. Additionally, this study adopted a standard FI development procedure (Searle et al., 2008; Theou et al., 2023; Zhao et al., 2023), ensuring an appropriately normative process for creating the PFI. Finally, the PFI can be generalized to similar contexts.

This study has some limitations. The first limitation is related to the selection of potential variables and there were no objective variables. However, psychological frailty is too complex to be described sufficiently with any objective variable and currently lacks a consistent definition (Fitten, 2015; Zhao et al., 2023), which explains the lack of criteria for selecting variables and different interpretations. Researchers have different interpretations of whether certain variables are the attributes of psychological frailty (Fitten, 2015; Kelaiditi et al., 2013; van Oostrom et al., 2017). Therefore, selecting variables related to psychological frailty is challenging. The second limitation is that the cut-off value yielded by the Youden index is debatable because no objective psychological frailty metric exists. The third limitation is that the TFI and 10-item CES-D were utilized as criteria for reliability analysis. Criterion-related reliability may be over- or underestimated owing to the utilization of these two criteria because they are imperfect as the standards of psychological frailty; in this study, they were considered as “quasi-gold” standard alternatives. Perfect identification is challenging because the definition and measurement criteria for psychological frailty currently lack consistency (Zhao et al., 2023). However, the selection of these two criteria facilitated the findings of both theoretical and practical significance. Fourth, the conceptual framework of psychological frailty that we have developed may lack breadth. As a domain of multidimensional frailty, psychological frailty could have both psychological and frail attributes (Zhao et al., 2023). Therefore, we defined psychological frailty as the coexistence of psychological problems and physical vulnerability (frailty). Most definitions or descriptions of psychological frailty in existing research have adopted a multidimensional framework (Lameirinhas et al., 2024; Zhao et al., 2023), which can and has led us to consider multidimensional attributes when operationalizing the term. Establishing a conceptual framework for psychological frailty is challenging and complicated due to the variety of psychological problems. Coupled with the fact that our choice of variables may not be complete due to the variable limitations of the CHARLS database, our conceptual framework may not be comprehensive enough. Fifth, we include physical constructs/vulnerability in conceptualizing psychological frailty based on previous literature. Shimada et al. (2019) have integrated physical frailty into the conceptual framework of psychological frailty. The mind–body interactions also support the notion that physical construct/vulnerability is an important component of psychological frailty (Fitten, 2015; Zhao et al., 2023). However, the absence of a consensus and extensive testing of this conceptual framework necessitates further research and validation. Sixth, although our study provides initial insights into the development of the PFI, it is significant to recognize its limitations related to validation; for this reason, our validation efforts should be considered preliminary. Seventh, the shift in response levels of lower life expectancy may have resulted in some loss of information; however, our ordinal regression analyses showed that the findings were still in the same direction. Eighth, our study used the CFA to validate the factor structure of psychological frailty for the total sample. However, given gender differences in the prevalence of psychological frailty, the factor structure might not apply equally to men and women. As such, we conducted additional analyses to examine cross-gender validity, and the CFA results support the stability of the factor structure across genders. Finally, the cross-sectional design of this study did not facilitate the examination of time sequences, precluding the determination of causal associations.

### 4.3 Future directions and implications

The PFI provides a new approach for classifying psychological frailty, opening avenues for subsequent research to examine its manifestations across diverse contexts. This study offers insights particularly into the operationalization of psychological frailty, its multidimensional attributes, and its interaction with physical vulnerability, which may serve as a foundation for future theoretical and empirical studies.

## 5 Conclusion

This study generated and partially validated a novel measurement instrument for assessing psychological frailty (a 15-item PFI) using CHARLS Wave 4 data. The findings highlight the potential capability of the PFI to predict adverse health outcomes. Although this study revealed good psychometric properties of the PFI, further research is needed to refine the validation process and enhance the interpretability of the PFI across diverse cohorts.

### What is already known?

- Psychological frailty is currently measured in two approaches: (1) the psychological domains of the multidimensional frailty instruments and (2) the combinations of the Fried frailty phenotype and psychological assessments.
- There is a lack of a measurement tool that specifically addresses psychological frailty.

### What this paper adds?

- A tailored measurement tool for psychological frailty was developed, which can have positive implications for a clear understanding of psychologically frail adults.
- Added a new measurement framework for psychological frailty that may offer potential value in reaching a consistent definition and measurement of psychological frailty.
- A positive association of psychological frailty with subjective life expectancy, outpatient treatments, and hospitalization was found in the CHARLS sample.

## Data Availability

Publicly available datasets were analyzed in this study. This data can be found here: all data were sourced from the China Health and Retirement Longitudinal Study (CHARLS).
