# Supplementary material for: Development of a psychological frailty index: results from the China health and retirement longitudinal study
Source: Front Psychol. 2025 Feb 11;16:1495733. doi: 10.3389/fpsyg.2025.1495733 (PMC11850362; doi:10.3389/fpsyg.2025.1495733)
Supplement: Supplementary file 1 [file Data_Sheet_1.docx]

**Supplementary material**

This material accompanies this article.

**Development and validation of a psychological frailty index: results from the China Health and Retirement Longitudinal Study (CHARLS)**

Authors: Jinlong Zhao, Justina Yat Wa Liu, Daniel Fernández, and Stefanos Tyrovolas

**Table of contents:**

[**Supplementary Figure 1** Flowchart of the study population 2](#_Toc167181470)

[**Supplementary Table 1** List of Potential Variables Associated with the Psychological Frailty Index 3](#_Toc167181471)

[**Supplementary Table 2** Variables Used for Creating the Tilburg Frailty Indicator 10](#_Toc167181472)

[**Supplementary Table 3** Descriptive Statistics by Sex and Psychological Frailty 11](#_Toc167181473)

[**Supplementary Figure 2** Confirmatory Factor Analysis Model of the Psychological Frailty Index 12](#_Toc167181474)

[**Supplementary Figure 3** Classification Accuracy Tests of the Psychological Frailty Index using Receiver Operating Characteristic Curves 13](#_Toc167181475)

# **Supplementary Figure 1** Flowchart of the study population

Participants enrolled in Wave 4

*N*= 19,817

With data related to psychological frailty variables

*n* =7,680 (38.75% of total)

Study participants (Total sample)

*n* =3, 934 (19.85% of total)

Missing values of 5% or more in psychological frailty variables

*n* = 719

Participants who responded *“DK,”“RF,” or “not assessed”

*n* = 2, 919

Missing outcome variables

*n* = 108

Exclusion criteria based on age

*n* = 12,137

Random sample

*n* =1, 290

(33.33% of the total sample derived by random sampling)

**Supplementary Figure 1** Flowchart of the study population

*DK: do not know; RF: refuses to answer

# **Supplementary Table 1** List of Potential Variables Associated with the Psychological Frailty Index

|  | | | | |
| --- | --- | --- | --- | --- |
| **Item** | **Variable name** | **Definition** | **Categories** | **Coding in PFI item** |
| PFI1 | DC011 | Depression: I felt depressed | rarely or none of the time (<1 day), some or a little of the time (1-2 days), occasionally or a moderate amount of the time (3-4 days), most or all of the time (5-7 days) | 0, 0.5, 0.75, 1, respectively |
| PFI2 | DC014 | Fearfulness: I felt fearful | rarely or none of the time (<1 day), some or a little of the time (1-2 days), occasionally or a moderate amount of the time (3-4 days), most or all of the time (5-7 days) | 0, 0.5, 0.75, 1, respectively |
| PFI3 | DC016 | Sadness: I was sad ^1^ | rarely or none of the time (<1 day), some or a little of the time (1-2 days), occasionally or a moderate amount of the time (3-4 days), most or all of the time (5-7 days) | 0, 0.5, 0.75, 1, respectively |

| **Supplementary Table 1** (Continued) | | | | |
| --- | --- | --- | --- | --- |
| **Item** | **Variable name** | **Definition** | **Categories** | **Coding in PFI item** |
| PFI4 | DC001_W4 | Disorientation: checking year | correct, error | Categorized 0/1 |
| PFI5 | DC002_W4 | Disorientation: checking season | correct, error | Categorized 0/1 |
| PFI6 | DC006_W4 | Disorientation: checking month | correct, error | Categorized 0/1 |
| PFI7 | DC007_W4 | Disorientation: checking the state | correct, error | Categorized 0/1 |
| PFI8 | DC008_W4 | Disorientation: checking county | correct, error | Categorized 0/1 |
| PFI9 | DC009_W4 | Disorientation: checking city | correct, error | Categorized 0/1 |
| PFI10 | DC016_W4 | Mild language impairment: watch correct | correct, error | Categorized 0/1 |
| PFI11 | DC017_W4 | Mild language impairment: pencil correct | correct, error | Categorized 0/1 |
| PFI12 | DD043_W4 | Thinking or understanding ability becomes much worse | no, yes | Categorized 0/1 |
| PFI13 | DC010 | Attentional problem: I had trouble keeping my mind on what I was doing | rarely or none of the time (<1 day), some or a little of the time (1-2 days), occasionally or a moderate amount of the time (3-4 days), most or all of the time (5-7 days) | 0, 0.5, 0.75, 1, respectively |
| PFI14 | DC004 | Self-described memory decline | excellent, very good, good, fair, poor | 0 =excellent; 0.25 =very good; 0.5 =good; 0.75 =fair; 1 =poor |

| **Supplementary Table 1** (Continued) | | | | |
| --- | --- | --- | --- | --- |
| **Item** | **Variable name** | **Definition** | **Categories** | **Coding in PFI item** |
| PFI15 | DD045_W4 | Amnesia: forgot what happened the day before today | no, yes | Categorized 0/1 |
| PFI16 | DD046_W4 | Amnesia: forgot where he is | no, yes | Categorized 0/1 |
| PFI17 | DD012_W4 | Memory impairment: recognize faces of families and friends | much better, improved a little, not much changed, a little worse, much worse | 0, 0.25, 0.5, 0.75, 1, respectively |
| PFI18 | DC009 | Anxiety: bothered by things | rarely or none of the time (<1 day), some or a little of the time (1-2 days), occasionally or a moderate amount of the time (3-4 days), most or all of the time (5-7 days) | 0, 0.5, 0.75, 1, respectively |
| PFI19 | DC013 | Negative coping: I cannot feel hopeful about the future ^2^ | rarely or none of the time (<1 day), some or a little of the time (1-2 days), occasionally or a moderate amount of the time (3-4 days), most or all of the time (5-7 days) | 0, 0.5, 0.75, 1, respectively |

| **Supplementary Table 1** (Continued) | | | | |
| --- | --- | --- | --- | --- |
| **Item** | **Variable name** | **Definition** | **Categories** | **Coding in PFI item** |
| PFI20 | DC017 | Loneliness: I felt lonely | rarely or none of the time (<1 day), some or a little of the time (1-2 days), occasionally or a moderate amount of the time (3-4 days), most or all of the time (5-7 days) | 0, 0.5, 0.75, 1, respectively |
| PFI21 | DC028 | Life dissatisfaction | completely satisfied, very satisfied, somewhat satisfied, not very satisfied, not at all satisfied | 0, 0.25, 0.5, 0.75, 1, respectively |
| PFI22 | DC042_W3 | Health dissatisfaction | completely satisfied, very satisfied, somewhat satisfied, not very satisfied, not at all satisfied | 0, 0.25, 0.5, 0.75, 1, respectively |
| PFI23 | DC015 | Sleeplessness: my sleep was restless | rarely or none of the time (<1 day), some or a little of the time (1-2 days), occasionally or a moderate amount of the time (3-4 days), most or all of the time (5-7 days) | 0, 0.5, 0.75, 1, respectively |

| **Supplementary Table 1** (Continued) | | | | |
| --- | --- | --- | --- | --- |
| **Item** | **Variable name** | **Definition** | **Categories** | **Coding in PFI item** |
| PFI24 | DA041_W4 | Psychosomatic pain | none, a little, somewhat, quite a bit, very | 0 =none, 0.25 =a little, 0.5 =somewhat, 0.75 =quite a bit, 1 =very |
| PFI25 | DC012 | Powerlessness: I felt everything I did was an effort | rarely or none of the time (<1 day), some or a little of the time (1-2 days), occasionally or a moderate amount of the time (3-4 days), most or all of the time (5-7 days) | 0, 0.5, 0.75, 1, respectively |
| PFI26 | DC018 | Emotional exhaustion: I could not ‘get going’ | rarely or none of the time (<1 day), some or a little of the time (1-2 days), occasionally or a moderate amount of the time (3-4 days), most or all of the time (5-7 days) | 0, 0.5, 0.75, 1, respectively |
| PFI27 | DB001 | limited physical activity: difficulty with running or jogging about 1km | do not have any difficulty, have difficulty but can still do it, have difficulty and need help, cannot do it | 0, 0.5, 0.75, 1, respectively |

| **Supplementary Table 1** (Continued) | | | | |
| --- | --- | --- | --- | --- |
| **Item** | **Variable name** | **Definition** | **Categories** | **Coding in PFI item** |
| PFI28 | DB004 | Physical exhaustion: difficulty with getting up from a chair | do not have any difficulty, have difficulty but can still do it, have difficulty and need help, cannot do it | 0, 0.5, 0.75, 1, respectively |
| PFI29 | DB007 | Functional limitations (limitations in stretching) | do not have any difficulty, have difficulty but can still do it, have difficulty and need help, cannot do it | 0, 0.5, 0.75, 1, respectively |
| PFI30 | DB008 | Decreased strength | do not have any difficulty, have difficulty but can still do it, have difficulty and need help, cannot do it | 0, 0.5, 0.75, 1, respectively |
| PFI31 | DD013_W4 | Memory loss: remember names of families and friends | much better, improved a little, not much changed, a little worse, much worse | 0, 0.25, 0.5, 0.75, 1, respectively |
| PFI32 | DD015_W4 | Remember things happened recently | much better, improved a little, not much changed, a little worse, much worse | 0, 0.25, 0.5, 0.75, 1, respectively |

| **Supplementary Table 1** (Continued) | | | | |
| --- | --- | --- | --- | --- |
| **Item** | **Variable name** | **Definition** | **Categories** | **Coding in PFI item** |
| PFI33 | DD016_W4 | Retrieval impairment: recall conversations a few days before | much better, improved a little, not much changed, a little worse, much worse | 0, 0.25, 0.5, 0.75, 1, respectively |
| PFI34 | DD022_W4 | Declining adaptive capacity: able to adapt to changes in daily life | much better, improved a little, not much changed, a little worse, much worse | 0, 0.25, 0.5, 0.75, 1, respectively |
| PFI35 | DD033_W4 | Impaired decision-making capacity: make decisions on everyday matters | much better, improved a little, not much changed, a little worse, much worse | 0, 0.25, 0.5, 0.75, 1, respectively |
| Note: 1. The variable DC016, whose original item was “I was happy,” was changed to “I was sad” based on the purpose of this study, and the evaluation method was the reverse scoring of the original item. | | | | |
| 2. The variable DC013, whose original item was “I felt hopeful about the future,” was adjusted to “I cannot feel hopeful about the future” based on the purpose of this study, and the evaluation method was the reverse scoring of the original item.  PFI: psychological frailty index. | | | | |

# **Supplementary Table 2** Variables Used for Creating the Tilburg Frailty Indicator

|  | | |
| --- | --- | --- |
| Items | Variable names | Variables from CHARLS |
| 1 | Self-Reported Health Status | DA002 |
| 2 | N/A | N/A |
| 3 | Difficulty with Running or Jogging about 1km | DB001 |
| 4 | Difficulty with Reaching or Extending your arms | DB007 |
| 5 | Hearing Problem | DA005_4_ |
| 6 | Vision Problem | DA005_3_ |
| 7 | Difficulty with Lifting or Carrying Weights over 10 Jin | DB008 |
| 8 | Difficulty with Getting up from a Chair | DB004 |
| 9 | Self-Rated Memory | DC004 |
| 10 | Felt Depressed | DC011 |
| 11 | Bothered by Things | DC009 |
| 12 | I Felt Everything I Did Was An Effort | DC012 |
| 13 | Have a Partner Living Together | BE002 |
| 14 | I Felt Lonely | DC017 |
| 15 | Any Possible Helper in the Future | DB030 |
| Note: N/A implies not available. In Wave 4, no data on weight is available. | | |

# **Supplementary Table 3** Descriptive Statistics by Sex and Psychological Frailty

|  | | | | | |
| --- | --- | --- | --- | --- | --- |
|  | **Total** | **Female** | | **Male** | |
|  | (*n*=3,934) | Non-psychological frailty | Psychological frailty | Non-psychological frailty | Psychological frailty |
|  |  | (*n*=826) | (*n*=1,000) | (*n*=1,478) | (*n*=630) |
| **Age** | | | | | |
| Mean (SD) | 71.04 (5.24) | 70.32 (4.92) | 71.10 (5.28) | 71.01 (5.18) | 71.95 (5.58) |
| **Financial status: additional income^a^** | | | | | |
| Not having | 3,548 (90.2%) | 775 (93.8%) | 959 (95.9%) | 1,232 (83.4%) | 582 (92.4%) |
| Having | 386 (9.8%) | 51 (6.2%) | 41 (4.1%) | 246 (16.6%) | 48 (7.6%) |
| **Highest education qualification^b^** | | | | | |
| Level 1 | 1,839 (46.7%) | 399 (48.3%) | 735 (73.5%) | 403 (27.3%) | 302 (47.9%) |
| Level 2 | 1,081 (27.5%) | 219 (26.5%) | 172 (17.2%) | 504 (34.1%) | 186 (29.5%) |
| Level 3 | 931 (23.7%) | 189 (22.9%) | 87 (8.7%) | 517 (35.0%) | 138 (21.9%) |
| Level 4 | 83 (2.1%) | 19 (2.3%) | 6 (0.6%) | 54 (3.7%) | 4 (0.6%) |
| **Total number of chronic conditions** | | | | | |
| Mean (SD) | 2.65 (1.98) | 2.39 (1.81) | 3.23 (2.12) | 2.17 (1.75) | 3.23 (2.11) |
| **Physical disability** | | | | | |
| Not having | 3,500 (89.0%) | 784 (94.9%) | 865 (86.5%) | 1,356 (91.7%) | 495 (78.6%) |
| Having | 434 (11.0%) | 42 (5.1%) | 135 (13.5%) | 122 (8.3%) | 135 (21.4%) |
| **Light physical activity^c^** | | | | | |
| Not meeting | 691 (17.6%) | 97 (11.7%) | 243 (24.3%) | 222 (15.0%) | 129 (20.5%) |
| Meeting | 3,243 (82.4%) | 729 (88.3%) | 757 (75.7%) | 1,256 (85.0%) | 501 (79.5%) |
| **Moderate physical activity^d^** | | | | | |
| Not meeting | 2,159 (54.9%) | 376 (45.5%) | 533 (53.3%) | 835 (56.5%) | 415 (65.9%) |
| Meeting | 1,775 (45.1%) | 450 (54.5%) | 467 (46.7%) | 643 (43.5%) | 215 (34.1%) |
| **Smoking status** | | | | | |
| Never smoked | 2,044 (52.0%) | 744 (90.1%) | 888 (88.8%) | 302 (20.4%) | 110 (17.5%) |
| Ever smoked | 1,890 (48.0%) | 82 (9.9%) | 112 (11.2%) | 1,176 (79.6%) | 520 (82.5%) |
| Note: As psychological frail were defined, those with PFI score ≥ 0.3083. a. Additional income refers to wage and bonus income received in the past year (excluding pensions). b. Highest education qualification: Level 1: Illiterate or did not complete primary school; Level 2: Home or primary school education; Level 3:Secondary school education included middle school, high school, and vocational school; Level 4; Higher education included two-/three-year college (or associate degrees), four-year college (or bachelor’s degrees), master’s degrees, and doctoral degrees. c. Light physical activity for at least 10 minutes at a time. d. Moderate physical activity for at least 10 minutes at a time. | | | | | |

# **Supplementary Figure 2** Confirmatory Factor Analysis Model of the Psychological Frailty Index


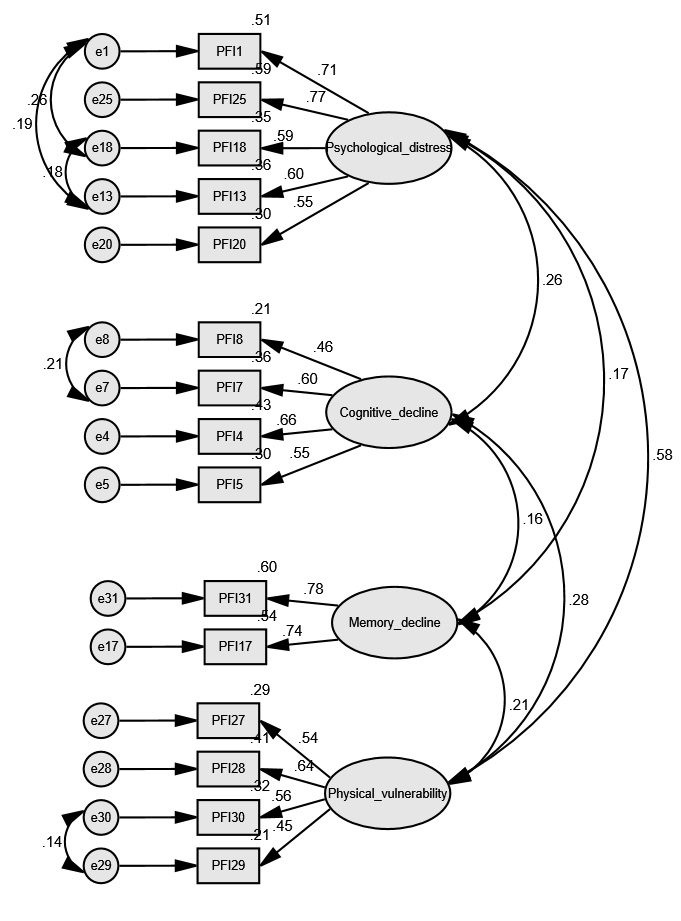


**Supplementary Figure 2** Confirmatory Factor Analysis Model of the Psychological Frailty Index

A correlation coefficient is represented using a double arrow, and a standardized regression coefficient using a path arrow. A box represents measuring items, and an ellipse represents a latent factor. All correlations between errors are significant with *p*<0.05. All regression coefficients are significant with *p*<0.001.

# **Supplementary Figure 3** Classification Accuracy Tests of the Psychological Frailty Index using Receiver Operating Characteristic Curves


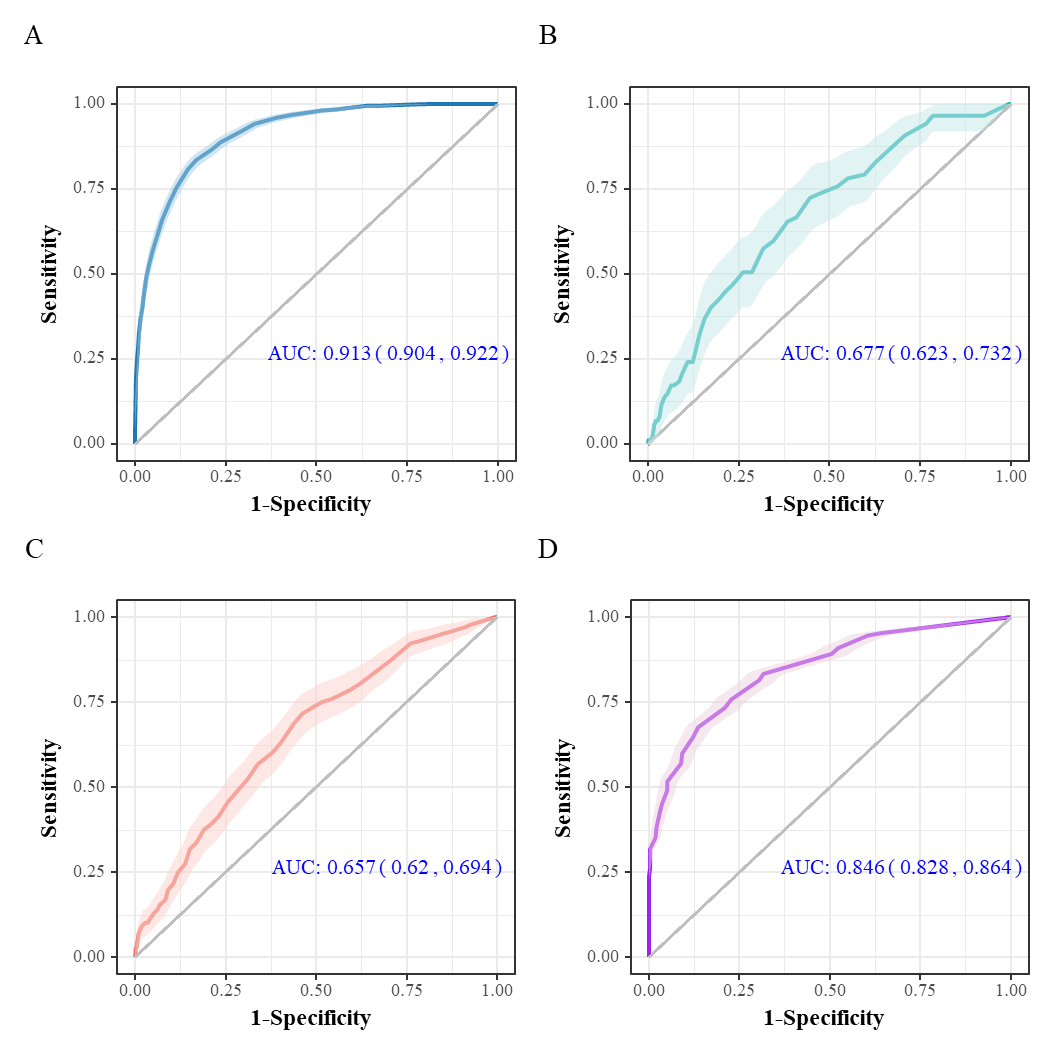


**Supplementary Figure 3** Classification Accuracy Tests of the Psychological Frailty Index using Receiver Operating Characteristic Curves

The AUC values with 95% CIs and the ROC curves with 95% CIs are presented. Graph **A**: the prediction of frailty (identified by TFI); Graph **B**: the prediction of diagnosed emotional or psychiatric problems; Graph **C**: the prediction of diagnosed memory-related diseases; Graph **D**: the prediction of depression (identified by 10-item CESD).
